# I. Hæmatosalpinx and Hæmatometra in a St. Bernard Dog. II. Pyosalpinx in a Dog; Autopsies

**Published:** 1895-09

**Authors:** 


### REPORTS OF CASES.

### I. HÆMATOSALPINX AND HÆMATOMETRA IN A ST. BERNARD DOG. II. PYOSALPINX IN A DOG; AUTOPSIES.

BY DR. SPENCER.

I. The dog, aged thirteen years, had had three litters of pups, the last about eight years before death. At the autopsy there was no peritonitis found, and no other disease than that shown in the uterus. The uterus and cornua were distended so as to be three to four centimetres in diameter. The cornua were greatly elongated, measuring 81 centimetres from one extremity to the other. The contained fluid weighed 12 ounces. Both cornua communicated freely with the body of the uterus, but no fluid escaped by the cervical canal into the ganglion. When pressure was made on the distended uterus the fimbriated extremities were closed by inflammation. The upper part of the cervix was blocked by a soft, sessile polypus, 5 millimetres in diameter, apparently growing from the mucous membrane. The fluid was viscid, about the consistency of molasses, and was dark red. Microscopic examination showed that in the wall of the cornua the mucous and submucous layers were replaced by inflammatory cells, and that the fluid consisted of débris and fat cells.

II. In this case also there was no peritonitis or other disease than that of the uterus and vagina. Both uterine cornua were distended with pus, so as to be about 3 centimetres in diameter and the wall was 2 to 3 millimetres in thickness. When pressure was made on the distended cornua no pus escaped into the uterus. The fimbriated ends were closed by inflammation and the pus had not a foul smell. The vagina contained pus and its mucous membrane was slightly inflamed. Microscopic examination showed that the inflammation of the cornua affected both mucous and submucous layers. The process probably extended from the vagina and then communication with the uterine cavity was shut off.

